# Transcript and Protein Profiling Analysis of the Destruxin A-Induced Response in Larvae of *Plutella xylostella*

**DOI:** 10.1371/journal.pone.0060771

**Published:** 2013-04-09

**Authors:** Pengfei Han, Fengliang Jin, Xiaolin Dong, Jiqiao Fan, Baoli Qiu, Shunxiang Ren

**Affiliations:** Engineering Research Center of Biological Control, Ministry of Education, South China Agricultural University (SCAU), Guangzhou, China; CHA University, Republic of Korea

## Abstract

**Background:**

Destruxins (dtxs) are the mycotoxin produced by certain entomopathogenic fungi, such as *Metarhizium anisopliae*, *Aschersonia* sp, *Alternaria brassicae* and *Ophiosphaerella herpotrichae.* It can affect a wide variety of biological processes in insects, including innate immune, Ca^2+^ channel in cells, and apoptosis in a dose-dependent manner. Dtxs have been used as biological control agent for a long time, however, their molecular mechanism of action is still unknown.

**Principal Findings:**

In this study, both digital gene expression (DGE) and two-dimensional electrophoresis (2-DE) approaches were adopted to examine the effects of dtx A on *Plutella xyllostella* (L.) larvae. By using DGE and 2-DE analyses, 1584 genes and 42 protein points were identified as being up- or down regulated at least 2-fold in response to dtx A. Firstly, injection of dtx A to larvae accelerated the increase of peptidoglycan recognition protein (PGRP), which could activate the Toll signal pathway inducing production of antibacterial substances such as cecropin and gloverin. Dtx A also stimulated prophenoloxidase (proPO) system which plays an important role in innate immunity and leads to melanization of external organisms. Secondly, dtx A suppressed the expression of genes related to the Toll pathway, and induced expression of serine proteinase inhibitors (serpins), especially the serpin 2 that blocked process of the proPO system. Finally, other physiological process like xenobiotics detoxification, apoptosis, calcium signaling pathway and insect hormone biosynthesis, were also mediated in response to dtx A toxicity.

**Conclusions:**

Transcript and protein profiling analyses will provide an insight into the potential molecular mechanism of action in *P. xylostella* larvae in response to dtx A.

## Introduction

Diamond back moth, *Plutella xylostella* (L.) (Lepidoptera: Plutellidae), is a mondial insect pest which threaten crucifer plant seriously, especially vegetables and oil seed crops. *P. xylostella* larvae feed on the plants from the seeding stage to harvest time influencing quality and yield of farm products. The damage caused by the insect results in significant losses and US $1.0 billion are spent globally on its management every year [1], [2]. Due to its high fecundity, overlapping generations and genetic plasticity, and selection pressure to various insecticides, *P. xylostella* has developed resistance to many insecticides [3], [4]. Biological control plays an important role in reducing pesticide residue and ensuring food safety. Entomopathogenic fungi have been used as biological control agents for a long time [5]. The fungi, like *Metarhizium anisopliae*, *Lecanicillium longisporum* and *Aschersonia* sp., are known for secreting 35 different types of dtxs [6], [7]. Among them, dtx A, B and E have shown insecticidal activities to wide range of insect pests such as *Spodoptera litura*, *Bemisia tabaci* [7]–[12]. Previous studies have shown that dtxs can suppress the hydrolytic activity of V-type ATPase in *Galleria mellonella*, cause oxidative stress in *Spodoptera litura*, influence the Ca^2+^ channel in muscle cells of *Manduca sexta* [13]–[15]. However, the potential molecular mechanism was not clearly understood based on genes and proteins.

Insects like other invertebrates have a potent and efficient innate immune system, which protects them from invading pathogens and parasites [16], [17]. When foreign invaders penetrate into the hemocoel, they are first recognized by recognition factors for cellular immune and humoral immune reactions [18]. After the recognition, modulating and signaling factors are stimulated, and signal transduction mainly included in Toll and Imd pathway is activated in specific tissues such as the fat body and hemocytes [19], [20]. Genes encoding effect molecules are activated through signaling cascades and a battery of these molecules, such as the antimicrobial peptides are produced in specific tissues and secreted into the hemolymph [16]. Recognition molecules induce activation of prophenoloxidase (proPO) system that leads to melanization of foreign organisms, wound healing, which is a defense mechanism against invaders in humoral immune system [21], [22]. Recent studies have suggested that dtxs can inhibit insect immune response [23], but this mechanism is still unknown.

Here, an expansive view of molecular mechanism of dtx A-induced toxicity response in *P. xylostella* was performed with the integrated bioinfomatics analysis of proteomic and transcriptomic data sets. In this context, the main objective of this study was to compare the gene expression pattern and protein profiles of *P. xylostella* between the control and the dtx A treatment at larval stage, identify potential genes and proteins mainly associated with toxicity response of insects. The transcript and protein profiling analyses will bring insight into the regulation of the toxicity response to dtx A in *P. xylostella.*

## Results

### Illumina Sequencing and Mapping DGE Tags to Transcriptome

**Figure 1:**
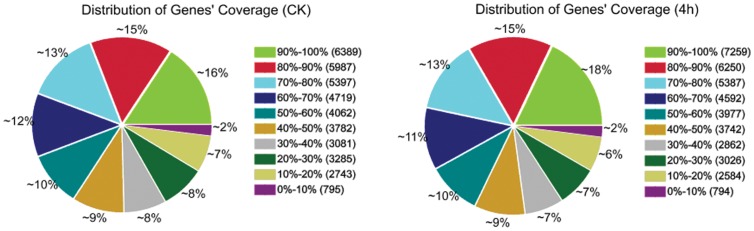
Distribution of genes’ coverage in each library.

Two DGE libraries of *P. xylostella* were sequenced including the treatment and control which generated between 7.16 and 7.53 million raw reads for each of the two libraries. After filtering the low quality reads, the total number of clean reads per library ranged from 7.06 to 7.44 million, and the percentage of clean reads in each library ranged from 98.68% to 98.75%. To evaluate whether the number of detected genes increases proportionally to total tag number, we performed the sequencing saturation analysis for the two samples. With the number of reads increasing, the number of detected genes was also increasing. However, when the number of reads reached 7.5 million, the growth rate of detected genes flattened, meaning that the number of detected genes tends to saturation. To assess comparability of DGE data, we analyzed the distributions of genes’ coverage. The distributions of genes’ coverage were similar, ensuring the comparability of genes between the control and treatment (Fig. 1).

To excavate the biomolecular information of *P. xylostella* response to dtx A, the reads sequences of the two DGE libraries were mapped to the reference transcriptome database (not revealed yet) generated using Illumina sequencing. Among the 7061594 and 7439232 clean reads generated from Illumina sequencing of the two libraries, 4747568 (67.23%) and 4963130 (66.72%) clean reads were mapped to the reference transcriptome database. The perfect matched reads were 2944970 (41.70%) and 2987334 (40.16%) respectively. Reads mapped to a unique sequence were the most critical subset of DGE libraries which can identify a transcript precisely. Ranged from 4377776 (58.85%) to 4191700 (59.63%) of the reads were explicitly identified and matched by unique tag (Table 1). All of above results indicated the reliability and operational stability of our experiment.

**Table 1:** Statistics of mapping to reference transcriptome for each library.

Map to gene	Control		Treatment	
	Reads number	Percentage	Reads number	Percentage
Total Reads	7061594	100%	7439232	100%
Total Base Pairs	346018106	100%	364522368	100%
Total Mapped Reads	4747568	67.23%	4963130	66.72%
Perfect match	2944970	41.70%	2987334	40.16%
≤2 bp mismatch	1802598	25.53%	1975796	25.56%
Unique match	4191700	59.36%	4377776	58.85%
Multi-position match	555868	7.87%	585354	7.87%
Total Unmapped Reads	2314026	32.77%	2476102	33.28%

### Expression Pattern Analysis

To identify and compare differentially expressed genes between the two libraries, the expression level of target genes were calculated with normalizing the number of unambiguous tags in each library to reads per kb per million reads (RPKM). To check whether the dtx A could cause significant changes in gene expression, we performed differential DGE analysis among the two groups using method Bayesian algorithm. The false discovery rate (FDR) ≤0.001 and absolute value of log_2_Ratio ≥1 were adopted as the thresholds to determine significant differences in gene expression. The results revealed that the expression level of 1,584 genes were significantly different between the control and treatment libraries. Among them, 674 and 910 genes were up-regulated and down-regulated respectively. We performed functional annotation of differential expression genes according to assign all genes to Nr database, Go database and KEGG pathway database. We compared differential expression genes with the whole transcriptome database background in order to search for genes mainly related to toxicity response of insects. Among the two DGE libraries, we chose annotated genes associated with toxicity response that mainly contained innate immune response, xenobiotics detoxification, calcium signaling pathway and apoptosis (Table S1).

Genes involved in recognition, including peptidoglycan recognition protein (PGRP), scavenger receptor, lectin, were dramatically up-regulated with the stress of dtx A. Among them, the PGRP showed the highest fold expression level (above 10 fold). In the category of signal transduction, differential expression genes in the Toll and Imd pathway were found such as toll, spatzle, cactus, relish and stat. After treatment with dtx A, the spatzle 6 precursor and spatzle 6 genes were all up-regulated 10 (log_2_), cactus and dorsal interacting protein were down-regulated, that indicated dtx A could inhibited Toll pathway. Lots of signal modulation related genes expressed differentially, that mainly contained mitogen activated protein kinase kinase kinase 5, 5-hydroxytryptamine receptor, TNF receptor associated factor, serine protease, and clip domain serine protease and serine protease inhibitor. 70% of these genes were serine protease that changed up-regulated to 11.25 and down-regulated to −11.09. Among these serine proteases, 19 genes were up-regulated and 11 down-regulated. Serpins hold 21 percentage of signal modulation related genes, in which only one gene was down-regulated. At the same time, mitogen activated protein kinase kinase kinase was up-regulated 10.14, 5-hydroxytryptamine receptor was down-regulated −10.08. That indicated serine proteases and serpins were main factors affected by dtx in the signal modulation of immune response. The immune effect system genes also expressed differentially with the injection of dtx A. Antibacterial peptide cecropin, gloverin, lysozyme, hemolymph proteinase were all up-regulated, while prophenoloxidase activating proteinase were down-regulated. With response to dtx A, carboxypeptidase B was down-regulated 12 and cadherin down-regulated 11.

Several xenobiotics detoxification genes, including cytochrome P450, Glutathione S-transferase, UDP-glycosyltransferase were up-regulated with dtx A treatment. Moreover, genes related to apoptosis such as apoptosis-inducing factor were up-regulated. Calcium signaling pathway and insect hormone biosynthesis genes containing calmodulin, mitochondrial ADP/ATP carrier protein, plasmic reticulum-type calcium ATPase, ryanodine receptor, juvenile hormone epoxide hydrolase and carboxylesterase were down-regulated in response to dtx A. We also observed that 70% of immune related genes have not expressed differentially (log_2_Ratio<1) in the digital expression profiling (data not showed).

### qRT-PCR Validation of DGE Consequence

**Figure 2:**
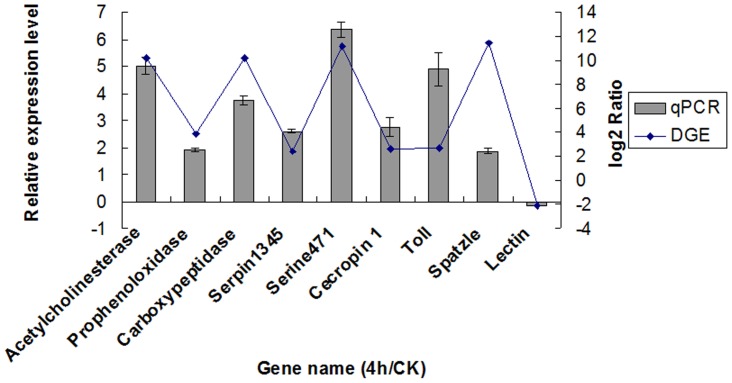
qRT-PCR validation of DGE result. Note: The left y-axis indicates the relative expression level by qRT-PCR, and the right y-axis indicates the log_2_Ratio of 4H/CK by DGE.

In order to confirm the quality of the DGE, real time qRT-PCR was carried out. Nine genes were randomly selected including Acetylcholinesterase, Prophenoloxidase Carboxypeptidase, Serpin1345, Serine471, Cecropin 1, Toll and Spatzle from the DGE analyses. The PCR products were notarized by sequencing and blasting in NCBI database. Quantification of the signals showed that all the patterns of gene expression were consistent with the DGE results, although the ratios varied to some extent (Fig. 2).

### Proteomic Analysis of *P. xylostella* Responses to dtx A

**Figure 3:**
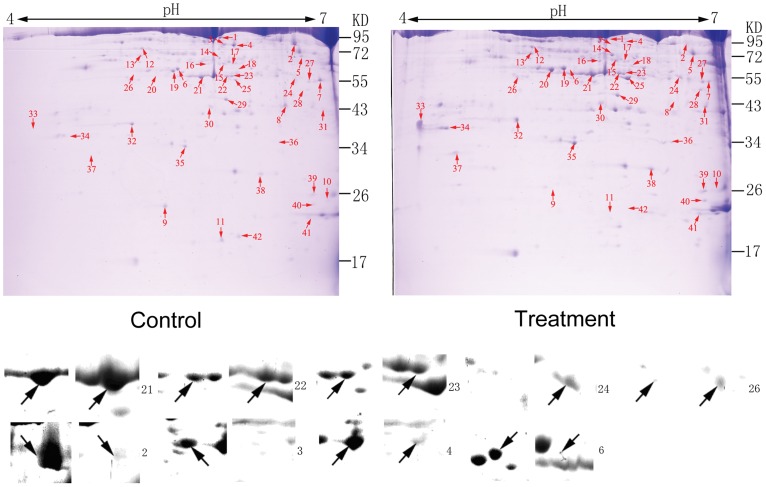
Two-dimensional electrophoresis map of hemolymph proteins. Note: 4^th^ instar larvae of *Plutella xylostella* were treated with destruxin A for 4 hour. Hemolymph proteins (1 mg) were separated on 2D gels (pH 4–7) and stained with Coomassie Brilliant Blue R-250. The differential expression and successfully identified protein spots are number, corresponding to the number in Table S2.

**Figure 4:**
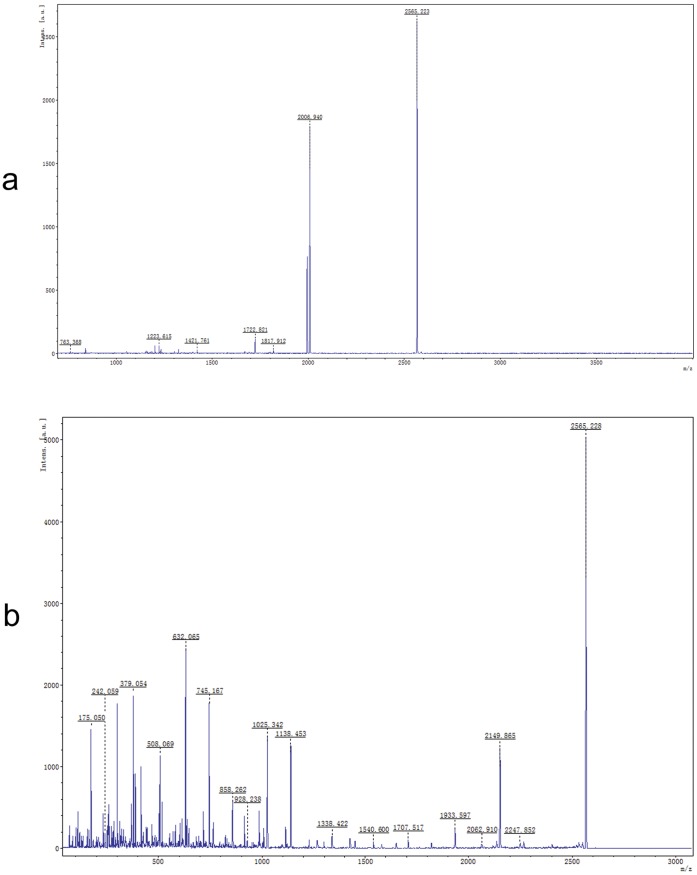
The MALDI-TOF/TOF-MS/MS analysis of protein spot 22. Note: The MALDI-TOF-MS peptide mass fingerprint spectrum of trypsin-digested protein (a) and its MS/MS peptide mass fingerprint spectrum of ionic peak 2565.23 (b).

After 4 h of injection, the insect was collected and the total hemolymph protein was extracted. Approximately 200 protein spots were detected on the gels and 126 spots were significantly different (fold ≥2) between the treatment and control gels. Finally 42 protein spots were successfully identified by using MALDI-TOF/TOF MS/MS on the basis of peptide mass matching. These differential spots were marked at each corresponding position (Fig. 3). Fig. 4 shows the presence of active spectrum of the trypsin digest of protein spots 22. The list of identified proteins is shown in Table S2 with their related information. After treatment with dtx A for 4 h, seven proteins were not present but ten new proteins appeared, simultaneously, 22 proteins were up-regulated and 4 proteins down-regulated. It is interesting that some protein spots were indentified as the same proteins including hexamerin-1, hexamerin-2, serpin-2, trypsin enzyme and homocysteine hydrolase. Locations of these spots in gels had difference in PI and molecular mass, indicated that they might have different post-translational modified. These identified proteins were classified into functional categories as follows: growth and development related proteins (hexamerin-1, hexamerin-2), metabolism related proteins (ATP synthase subunit beta, mitochondrial, S-adenosy1-L-homocysteine hydrolase, arginine kinase, glucosinolate sulfatase, enolase, protein Y43F4B.5), cytoskeleton proteins (actin, actin-depolymerizing factor 1), toxicity response related proteins (serpin 1b, serpin 2, cathepsin L-like cysteine proteinase, trypsin-like enzyme, protein spaf-1718, glutathione S transferase 2-like protein) and other proteins. Approximately 26% of these proteins were involved in toxicity response and up-regulated, in which five serpin-2 and one serpin-1b were identified. At the same time, many hexamerin proteins were down-regulated, which were involved in development.

### Western Blot Confirmation for Differentially Expressed Protein

**Figure 5:**
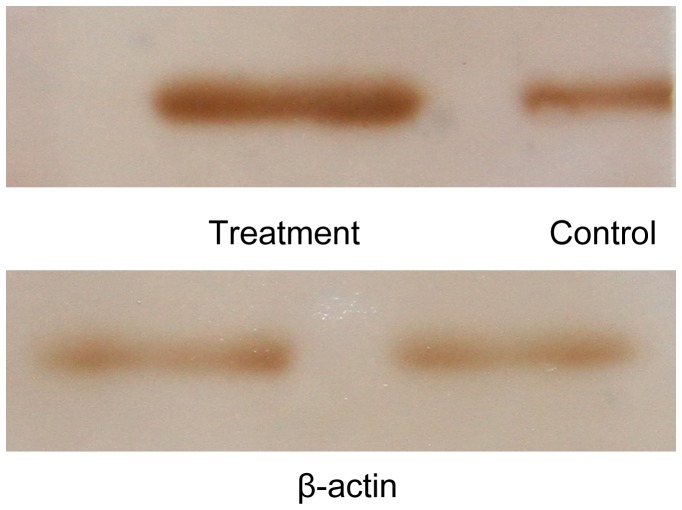
Western blot analysis of expression of *Px*Serpin 2. Note: Those were visualized by DAB. Actin was used as an internal control.

To determine whether the expression of *Px*Serpin 2 protein increased under the dtx A stress, western-blotting analysis was performed. Immunoblot analysis detected *Px*Serpin 2 protein with a MW of ∼40 kDa (Fig. 5), the change of whose expression was consistent with that in transcript and protein profiling analysis, demonstrating that *Px*Serpin 2 protein was expressed more abundantly in response to dtx A.

## Discussion

Changes in mRNA levels do not always lead to similar alterations in protein levels. Our integration transcriptome and proteome analysis will provide an intensive insight into the potential molecular mechanism of the toxicity response to dtx A in *P. xylostella*.

Dtxs are cyclic peptide mycotoxins that has been implicated in the infection process of entomopathogenic fungi and have high toxicity to different insect species when ingested or injected [24], [10], [11], [25]. Studies have shown that dtx A could suppress the expression of various antimicrobial peptides in *Drosophila melanogaster* [23] and increase the virulence of entomopathogenic fungi (*Isaria javanicus*) to *P. xylostella* [26]. These studies suggested that *Metarhizium anisopliae* or dtx A may be an effective insecticide. In this study, LC_50_ for 4^th^ larvae treated with dtx A after 24 h was 200 µg/mL, this concentration will be used in future research.

In this study, we have generated an integrated *P. xylostella* transcriptome dataset (not revealed yet) using high throughput sequencing Illumina, which is important for getting the details of bioinformation especially when the genomic information of *P. xylostella* is unavailable. Subhamoy (2007) used the microarray sequencing to analyze the changes of mRNA in *Drosophila melanogaster* with the treatment of dtx A. Compared with microarray technology, the next-generation high-throughput Illumina sequencing technology provides millions of sequence reads in a single run. This capacity promotes gene expression profiling experiment with an improved dynamic range and arrestive cost saving [27]. By using the DGE profiling method, we sequenced 7 million reads across the two cDNA libraries. Among them 2.9 million tags were successfully mapped to the reference transcriptome. Many genes and proteins with significantly differential expression were related to innate immune response, xenobiotics detoxification, apoptosis, calcium signaling pathway (Table S1&S2).

Peptidoglycan recognition protein (PGRP) that promotes immunity is well recognized in *Drosophila melanogaster* and other insect species [28]. Under the pressure of dtx A, PGRP was significantly up-regulated (Table S1), the scavenger receptor and C-type lectin were also up-regulated simultaneously. But the nicotinic acetylcholine receptor was down-regulated in this process. C-type lectins have been well studied for the roles in non-self recognition, immune signaling transduction [29]. A fungal metabolite asperparaline A from *Aspergillus japonicus* JV-23 targets the nicotinic acetylcholine receptor (nAChR) among the ligand-gated ion channels expressed by *Bombyx mori* neurons, offering an explanation that this compound caused paralysis of silkworm larvae [30]. These results suggest that PGRP might be the potential receptor of dtx A in *P. xylostella*, and the target of dtx A may be the nicotinic acetylcholine receptors. In the events of dtx A recognition, spatzle included in the Toll pathway showed the highest expression. But dorsal interacting protein and cactus were down-regulated. These data indicated that dtx A could suppress gene expression in Toll pathway and meantime the insect could resist dtx A by Toll pathway.

Proteolytic activation of prophenoloxidase (proPO) system plays an important role in innate humoral immune. Phenoloxidase (PO) leads to melanization of foreign organisms, wound healing. Conversion proPO to PO is accomplished by limited proteolysis through a PO-activating cascade including activation of multiple serine proteases, whereas serpins blocked this process [22], [31], [32]. In this study, a large number of serine protease genes were up and down-regulated and lots of prophenoloxidase activating proteinases were up-regulated by DGE analysis (Table S1). In the result of 2-DE, Cathepsin L-like cysteine proteinase highly expressed for 45 fold (Table S2, Fig. 3) that affects the immune-related proteolytic activation cascade leading to production of active phenoloxidase. This process is negatively regulated by serpins in the haemolymph [33]. In the model species of *Manduca sexta*, serpins inhibit proteinases in the prophenoloxidase activation system, thereby regulating the response [34]. In our experiment almost all the serpins were up-regulated, especially the serpin 2 which appeared to be correlated in terms of their expression trends at both mRNA and protein levels (Table S1&S2, Fig. 3&5). Four same serpin 2 were identified in 2-DE analysis, this phenomenon might be the result of different post-translational modifications. These results revealed that the dtx A might induce the expression of serpins to suppress the proPO system.

Antimicrobial peptides (AMPs), which are produced by several species including insects and other animals, are a critical component of the natural defense system [35]. Several AMPs including cecropin, gloverin, and lysozyme were up-regulated (Table S1), dtx A could suppress innate humoral immune response by reducing the expression of various antimicrobial peptides in *Drosophila melanogaste* [23], but this trends was not identified in the tandem mass spectrometry. This phenomenon suggested that the dtx A suppressed the translation of the antimicrobial peptides genes. Hemolymph proteinase 5, 8, 9 were up-regulated under the stress of dtx A (Table S1). Hemolymph proteinase-6 is a component of both prophenoloxidase activation and Toll pathways in *Manduca sexta.* Serpin-5 regulates prophenoloxidase activation and the Toll signaling pathway by inhibiting hemolymph proteinase 6 [36]. Serpins may suppress hemolymph proteinase 6 induced by dtx A in *P. xylostella.* Both transcript and protein profiling analysis results showed that the hexamerin presented down-regulated trend after the treatment of dtx A and the hexamerin-1, hexamerin-2 performed post-transcriptional modification (Table S2). Hexamerins are synthesized in the fat body and secreted into the hemolymph, which provide a buffer between resource availability and metabolic need for processes such as molting, egg production, adult diapause [37]–[39]. The results suggested that dtx A might cause lack of amino acid to accomplish life history and finally death of the *P. xylostella.*

In response to dtx A, some enzymes related to the metabolism of xenobiotics differentially expressed, including glutathione S-transferase, cytochrome P450 and UDP-glycosyltransferase. Glutathione S-transferase gene was up-regulated in the proteomic analysis, and its mRNA levels displayed a similar expression trend (Table S1&S2). Previous reports have proved that cytochrome P450 monooxygenases and glutathione S-transferase facilitated the insect to tolerate or resist to some drugs, pesticides, and plant toxins [40], [41]. UDP-glycosyltransferase protects the cellular system from being damaged by toxic foreign compounds [42]. The results suggested that cytochrome P450, Glutathione S-transferase and UDP-glycosyltransferase played an important role in the detoxification of dtx A.

Cytosolic calcium (Ca^2+^) signals control many cellular functions from short-term responses such as contraction and secretion to long-term regulation of transcription, growth and cell division [43]. In our research, calcium signaling pathway-related genes identified in this study were down-regulated in response to dtx A showing that dtx A can influence calcium-dependent processes in insect, and might be acting on insect visceral muscle by facilitating an influx of extracellular Ca^2+^ [44]. Meanwhile, the up-regulation of the apoptosis-inducing factor was consistent with report showing that dtx A can induce apoptosis of SL-1 cells in *Spodoptera litura* [45]. In insects juvenile hormone regulates both metamorphosis and reproduction [46]. Expression of enzymes related to metabolism of juvenile hormone was inhibited by dtx A (Table S1). Previous study showed that microsporidia could reduce activity of juvenile hormone esterase in *Lymantria dispar* larvae. This disturbance of juvenile hormone metabolism caused delayed development and failure of successful pupation [47]. Dtx A is produced by certain entomopathogenic fungi. It is possible that dtx A influence development of insects by disturbing the metabolism of juvenile hormone.

Therefore, the toxicity of mycotoxin dtx A to *P. xylostella* was the results of various factors. There was also relationship of co-evolution between entomogenous fungi and insects. The gene expression profiles based on transcriptome and protein profiling provide an insight into the potential molecular mechanism of the toxicity response to dtx A in *P. xylostella*, which will play an important role in better application of entomopathogenic fungi and the new insecticide research for pest control.

## Materials and Methods

### Digital Gene Expression Profiling

#### *P. xylostella* strains and dtx A

The susceptible *P. xylostella* strain was collected from the Engineering Research Centre of Biological Control, Ministry of Education, South China Agricultural University, Guangzhou, Guangdong province, and maintained without exposure to insecticide for 10 generations. Rearing conditions were set at 25±1°C, 65% RH, a 14-h light/10-h dark photoperiod and 1000–1500 lx intensity. Dtx A was isolated and purified from strain MaQ10 of *Metarhizium anisopliae* [48]. The purity of dtx A was determined by high performance liquid chromatography (HPLC). It was then diluted with phosphate buffered saline (PBS, PH7.4).

#### Treatment schedule and sample preparation

The susceptible *P. xylostella* 4^th^ instar larvae were injected with 2 µl of a solution containing 200 µg/ml dtx A (LC_50_) with the microinjector. Control larvae were injected with PBS. After 4 hours of treatment, ten larvae were collected respectively and immediately frozen in liquid nitrogen. The total RNA was extracted using Trizol Total RNA Isolation Kit (Takara, Japan) according to manufacturer’s protocol. Quality and quantity were confirmed using Nanodrop (Bio-Rad, USA) and 2100 Bioanalyzer (Agilent, USA). Three biological replicates were arranged for each strain.

#### Construction of DGE library and Illumina sequencing

Tag library construction for the two samples (dtx A treated group and control group) was performed by using the Illumina Gene Expression Sample Prep Kit. Briefly, mRNA was isolated from total RNA with magnetic oligo (dT) beads. Taking that as templates, random hexamer-primers were used to synthesize first-strand cDNA. Second-strand cDNA was synthesized using buffer, dNTPs, RNaseH and DNA polymerase. And bead-bound cDNA was subsequently digested with the restriction enzyme *Nla* III that recognizes and cuts off the CATG sites. The cDNA fragments with 3′ ends were then purified with magnetic beads and the Illumina adapter 1 was added to their 5′ ends. The junction of Illumina adaptor 1 and CATG site is the recognition site of *Mme* I, which is a type of endonuclease with separated recognition sites and digestion sites. It cuts at 17 bp downstream of the CATG site, producing tags with adaptor 1. After removing 3′ fragments with magnetic bead precipitation, the Illumina adaptor 2 is ligated to the 3′ ends of tags, generating tags with different adaptors at both ends to form a tag library. After linear PCR amplification, 95 bp fragments are purified by PAGE Gel electrophoresis. After denaturation, the single-chain molecules are fixed onto the Illumina Sequencing Chip. Each molecule grows into a single-molecule cluster sequencing template through in situ amplification. Four types of nucleotides, which are labeled by four colors, are added and sequencing is performed using the sequencing by synthesis (SBS) method. Each tunnel generates millions of raw data with a sequencing length of 49 bp.

#### Mapping of DGE tags to *P. xylostella* transcriptome database

Raw images received from sequencing were transformed into sequence data. Ahead of mapping reads to transcriptome databases, we filtered all sequences to remove low quality tags, such as tags with unknown sequences ‘N’, empty tags (sequence with only adaptor sequences), low complexity tags, and tags with only one copy (most likely resulting from sequencing errors). A library containing all CATG+17 bases length tags was created with our transcriptome database. All tags were mapped to the reference sequences and allowed only one base mismatch. Clean tags that could map to reference sequences were filtered from multiple genes. The remainder of the clean tags was designed unambiguous clean tags. For gene expression analysis, the number of unambiguous clean reads for each gene was calculated and normalized to RPKM (Reads Per Kb per Million reads). These different expressed tags were used for mapping and annotation [49].

#### Analysis of differential expression genes

Statistical analysis of the frequency of each tag in the different cDNA libraries was performed to compare gene expression in both the treatment and control conditions. The statistical comparison was performed with custom written scripts using the method described by Audic [50]. FDR (False Discover Rate) was adopted to determine the threshold of the P value in multiple tests and analyses. We used FDR≤0.001 and the absolute value of log_2_Ratio≥1 as the threshold to judge the significance of gene expression differences [51]. We performed cluster analysis of gene expression patterns with the software of cluster and Java Treeview [52], [53]. In the DGE profiling analysis, Gene Ontology enrichment analysis of functional significance was implemented using the hypergeometric test to map all differentially expressed genes to terms in the GO database, looking for significantly enriched GO terms in differentially expressed genes, and comparing them to the reference transcriptome database. For the pathway enrichment analysis, we mapped all differentially expressed genes to terms in the KEGG database and looked for significantly enriched KEGG terms compared to the reference transcriptome database (not revealed yet).

### Two-dimensional Gel Electrophoresis (2-DE)

#### Treatment of the insects and protein preparation

After 4 hours of treatment that was same as the sample prepared for DGE, body surface of 4^th^ instar larvae were disinfected using 75% alcohol, and then prosected for hemolymph collection with capillary, the hemolymph was dissolved in PBS added proper protease inhibitor and Dithiothreitol (DTT). The mixture was centrifuged at 1200 g for 5 min at 4°C, supernate was transferred into another EP tubes. Proteins were extracted according to the methods described [54]. Added directly 10% trichloroacetic acid (TCA) in acetone containing DTT (0.2% W/V) into PBS solution and vortexed for 5 min. Furthermore, the proteins were allowed to precipitate over night at −20°C. Precipitated protein was centrifuged at 12,000 g for 25 min at 4°C. The precipitate was washed with cool pure acetone for two times (12,000 g, 15 min, 4°C), dried for about 5 min using vacuum drier and then dissolved with lysis buffer: 7 M Urea, 2 M Thiourea, 4% (w/v) CHAPS, 1%(w/v) DTT, 0.2% (w/v) pH 3–10 Ampholyte, 50 mg/mL RNase, 200 mg/mL DNase and 0.5% (v/v) protease inhibitors cocktail. The protein concentration was quantified according to the Bradford method [55].

#### First-dimension isoelectric focussing (IEF)

2-DE of hemolymph proteins of *P. xylostella* was performed using a 2-DE system (Bio-Rad, USA) according to the instructions. 400 µl of total protein (1 mg) diluted with lysis buffer was loaded in 17 cm, pH 4–7 IPG strips (Bio-Rad, USA) for isoelectric focusing. The IEF program as follows: active rehydrate at 20°C, 50 V for 12 h, a linearly increasing gradient from 0 to 100 V for 1 h, speediness increasing to 200 V for 0.5 h, linearly increasing to 1000 V for 0.5 h, linearly increasing to 4000 V for 1.5 h, speediness keeping 4000 V for 6000 Vh, and electric current for each strip limited to 50 µA.

#### Second-dimension SDS-PAGE and image analysis

Before the electrophoresis in the second dimension, the IPG strips were equilibrate continuously for 15 min with equilibration solution I (6 M urea, 0.375 M Tris-HCl, pH 8.8, 20% glycerol, 2% SDS and 20 mg/mL DTT) and then the equilibration solution II (25 mg/mL iodoacetamide instead of DTT). The equilibrated strips were run on 12% SDS-polyacrylamide gels at 10 mA per gel for 1 h and 50 mA per gel until the bromphenol blue (sealing the IPG gels with agarose sealing solution, containing 0.5% agarose, 0.1% SDS, 25 mM Tris-HCl, 0.001% bromophenol) front reached the bottom of the gel, and electrophoresis was performed at 18°C. This experiment was performed for three times. The proteins in gels were dyed with Coomassie Brilliant Blue R-250 solution (50% ethanol, 10% acetic acid, and 40% water) overnight. The gel images were scanned using Umax scanner and analyzed quantitively with PDquest version 8.0 software (Bio-Rad, USA). To get the detail of differential expression proteins, control gels were performed as reference to be compared with the treatment gels. Protein spots in which the differential expression index ≥2- fold compared with the control were chosen for further identification.

#### MALDI-TOF/TOF-MS/MS analysis for proteins and database search

The 42 differentially expressed proteins were excised manually from three replicate gels. The target spots were then digested with digestion solution (40 mM NH_4_HCO_3_ in 9% acetonitrile solution, and 20 µg/ml proteomics grade trypsin) into tryptic peptides that were analyzed by matrix-assisted laser desorption/ionization time-of-flight tandem mass spectrometry (MALDI-TOF MS/MS) with a mass spectrometer (Bruker Dalton, Germany). Flex Analysis (Bruker Dalton, Germany) and BioTools (Bruker Dalton, Germany) software were used to distinguish signal peak and search peptide and protein from NCBI databases. The search was performed as following settings: peptide mass range from 800–4000 Da, one missed cleavage, global modifications of carbamidomethy, variable modifications of oxidation, peptide tolerance 50 ppm, fragment mass tolerance 0.5 Da, peptide charge +1. The high scoring identified proteins were selected with expected P-values <0.05.

### Quantitative Real-time PCR (qRT-PCR) Validation

To confirm the digital expression profiling results, we have randomly chosen and designed 9 pairs of primer about innate immune response genes to perform qPCR analysis. The primers are shown in Table 2. Total RNA sample was extracted as described for the DGE experiment, which was the same biological replicates as DGE sample. The RNA was reverse transcribed in a 20 µl reaction system using the AMV RNA Kit (TaKaRa, Japan) according to the manufacturer’s protocol. The qPCR was performed using a BIO-Rad CFX-96 Real-Time PCR system with the iTaq Universal SYBR Green Supermix Kit (BIO-Rad, USA) according to the manufacturer’s protocol. The beta-actin gene was used to normalize the expression levels with that average threshold cycle (Ct) was calculated. Each reaction was run in triplicate and the relative expression of genes was calculated using the (2^−ΔΔCt^) method.

**Table 2:** The primers used in qRT-PCR.

Primers	Forward	Reserve
Acetylcholinesterase	TGCTACCAAGAGCGGTACGAGTA	CACCCATATGTTCAAATAGAGGC
Carboxylesterase	TGCTACCAAGAGCGGTACGAGTA	CACCCATATGTTCAAATAGAGGC
Prophenoloxidase	TTTTGATTGTGTGTGTTATGTGG	TTCTGTTGATAGCGAGGAGTGGC
Serpin 1345	CCACGATTCCAGTTTGATTACAC	GTTGACCTCGATACCAGCCTTCT
Serine	AGTCATAGCACGAAGATCCAACC	AAAACGAATCAATAAAGACCGCA
Cecropin 1	GTCGCTGTCATCGGACAAGCCAC	TATACATTATTTAACCCGTAAAT
Toll	TTTTTTGGGTCAACTGCGTAAAC	GCGTGAAACTCCATTGTCATAGC
Spatzle	ACTGCTAACAACCTGTGTGGAGA	CCGAGAGAGGAACTTGAGGGTCA
Lectin	GACACAGGAACAATTCGATATCT	GGCTGCTGACTCCGACCCAGGCC

### Western Blot

Western-blotting analysis was modified according to the methods from the previously described [56]. Briefly, hemolymph proteins were extracted from 4^th^ instar larvae of the *P. xylostella* in two different treatment groups. Totally 350 µg hemolymph proteins were separated on a 12% SDS-PAGE gel, which was semi-dry transferred for 25 min at 15 V to PVDF membrane (Bio-Rad, USA), immunoblotted with anti- *Px*Serpin 2 serum (diluted 1∶5000) and an IgG goat anti-rabbit antibody conjugated with HRP was used for secondary antibody (BOSTER, China, 1∶5000 dilution), finally visualized by DAB.

### Conclusion

In conclusion, this is a comprehensive study of transcriptomic and proteomic analyses on *P. xylostella* in response to dtx A. The results showed that dtx A was recognized by peptidoglycan recognition protein and inhibited the Toll signal pathway. Dtx A induced expression of serpins to suppress the proPO system. Dtx A influenced apoptosis, calcium signaling pathway and development of insect. This study contributes to the understanding of potential molecular mechanism of the toxicity response to dtx A in *P. xylostella.*

## Supporting Information

Table S1 Genes related to toxicity response by DGE.
(DOC)

Table S2 Identification of hemolymph proteins by MALDI-TOF/TOF-MS/MS.
(DOC)
