# Supplementary material for: Transcript and Protein Profiling Analysis of the Destruxin A-Induced Response in Larvae of Plutella xylostella
Source: PLoS One. 2013 Apr 9;8(4):e60771. doi: 10.1371/journal.pone.0060771 (PMC3621956; doi:10.1371/journal.pone.0060771)
Supplement: Table S1 — Genes related to toxicity response by DGE. (DOC) [file pone.0060771.s001.doc]

**Table S1. Genes related to toxicity response after treatment with dtx-A by DGE. Significantly different expressed genes between CK and 4H are based on FDR ≤ 0.001 and the absolute value of Log_2_Ratio ≥ 1. The Log_2_Ratio (4H/CK) indicates the change of gene e**xpression.

| **Gene** | **Length** | **RPKM-CK** | **RPKM-4H** | **Log2Ratio(4H/CK)** | **Description** |
| --- | --- | --- | --- | --- | --- |
| **Immune response** | | | | | |
| ***Recognition molecules*** | | | | | |
| gi|117970194| | 305 | 0.001 | 1.498 | 10.549 | Peptidoglycan recognition protein [*Plutella xylostella]* |
| gi|357631633| | 728 | 130.425 | 442.106 | 1.761 | Peptidoglycan recognition protein C [*Danaus plexippus*] |
| gi|357631632| | 1092 | 26.434 | 69.658 | 1.398 | Peptidoglycan recognition protein-D [*Danaus plexippus*] |
| gi|114050807| | 349 | 2.734 | 13.09 | 2.259 | Lectin 5 precursor [*Bombyx mori*] |
| gi|148298818| | 934 | 15.581 | 46.957 | 1.592 | C-type lectin 10 precursor [*Bombyx mori*] |
| gi|284813581| | 563 | 16.102 | 48.282 | 1.584 | C-type lectin 19 precursor [*Bombyx mori*] |
| gi|357628226| | 290 | 3.291 | 0.788 | -2.027 | Nicotinic acetylcholine receptor [*Danaus plexippus*] |
| gi|357624410| | 225 | 2.121 | 8.122 | 1.937 | Scavenger receptor class B [*Danaus plexippus*] |
| gi|91085301| | 2781 | 18.1 | 68.257 | 1.915 | Scavenger receptor class B [*Tribolium castaneum*] |
| ***Signal transduction*** | | | | | |
| gi|357617307| | 268 | 0.89 | 5.97 | 2.745 | Toll [*Danaus plexippus*] |
| gi|318104931| | 280 | 3.408 | 10.606 | 1.638 | Toll receptor 18 wheeler [*Spodoptera frugiperda*] |
| gi|357611178| | 294 | 6.492 | 15.539 | 1.259 | Putative toll [*Danaus plexippus*] |
| gi|357607081| | 952 | 3.759 | 7.918 | 1.075 | Putative toll [*Danaus plexippus*] |
| gi|282158077| | 395 | 0.001 | 1.157 | 10.176 | Spatzle 6 precursor [*Tribolium castaneum*] |
| gi|256274668| | 488 | 0.489 | 2.809 | 2.522 | Spatzle A [*Manduca sexta*] |
| gi|357606946| | 248 | 0.001 | 2.763 | 11.432 | Spatzle 6 [*Danaus plexippus*] |
| gi|289629214| | 1112 | 134.961 | 32.61 | -2.049 | Cactus [*Bombyx mori*] |
| gi|357614284| | 291 | 4.099 | 1.57 | -1.3845 | Dorsal interacting protein 3 [*Danaus plexippus*] |
| gi|346987771| | 3077 | 26.904 | 76.093 | 1.50 | Relish [*Helicoverpa armigera*] |
| gi|119351872| | 316 | 29.443 | 82.407 | 1.485 | STAT [*Hyphantria cunea*] |
| gi|357618121| | 264 | 0.904 | 5.192 | 2.522 | Signal transduction protein lnk-realted [*Danaus plexippus*] |
| ***Signal modulation*** | | | | | |
| gi|357605643| | 202 | 0.001 | 1.131 | 10.143 | Mitogen activated protein kinase kinase 5 [*Danaus plexippus*] |
| gi|2494933| | 221 | 1.079 | 0.001 | -10.076 | 5-hydroxytryptamine receptor |
| gi|357612388| | 347 | 3.438 | 1.317 | -1.385 | TNF receptor-associated factor 3 interacting protein 1 [*Danaus plexippus*] |
| gi|39655053| | 840 | 0.001 | 2.447 | 11.257 | Pattern recognition serine proteinase precursor [*Manduca sexta*] |
| gi|357623992| | 607 | 0.001 | 2.528 | 11.141 | Serine protease [*Danaus plexippus*] |
| gi|357606346| | 297 | 0.001 | 0.769 | 9.587 | Serine protease 120 [*Danaus plexippus*] |
| gi|63207765| | 1118 | 1.92 | 18.797 | 3.29 | Serine protease 1 [*Lonomia obliqua*] |
| gi|357617295| | 448 | 3.728 | 34.672 | 3.217 | Pattern recognition serine proteinase precursor [*Danaus plexippus*] |
| gi|208972545| | 523 | 12.316 | 75.123 | 2.609 | Serine proteinase-like protein 1 [*Helicoverpa armigera*] |
| gi|239048216| | 562 | 9.339 | 49.587 | 2.409 | Serine protease 22 precursor [*Nasonia vitripennis*] |
| gi|237700825| | 217 | 1.099 | 5.263 | 2.259 | Serine protease 46 [*Mamestra configurata*] |
| gi|208972549| | 1508 | 65.179 | 275.384 | 2.079 | Serine proteinase-like protein 1 [*Helicoverpa armigera*] |
| gi|270012759| | 553 | 4.314 | 16.936 | 1.973 | Serine protease P66 [*Tribolium castaneum*] |
| gi|357616872| | 1667 | 38.497 | 149.498 | 1.957 | Serine protease 7 [*Danaus plexippus*] |
| gi|56462344| | 774 | 17.877 | 57.254 | 1.679 | Serine proteinase-like protein 1 [*Lonomia obliqua*] |
| gi|357619638| | 316 | 5.285 | 15.903 | 1.589 | Serine protease-like protein precursor [*Danaus plexippus*] |
| gi|112983618| | 551 | 3.897 | 11.193 | 1.522 | Pattern recognition serine proteinase precursor [*Bombyx mori*] |
| gi|379699022| | 639 | 6.72 | 15.371 | 1.194 | Putative trypsin-like serine protease [*Bombyx mori*] |
| gi|357611015| | 2610 | 19.652 | 42.622 | 1.117 | Serine protease 33 [*Danaus plexippus*] |
| gi|304443615| | 321 | 2.973 | 6.404 | 1.107 | Serine proteinase-like protein precursor [*Mamestra configurata*] |
| gi|114052256| | 503 | 9.486 | 20.436 | 1.1 | serine proteinase-like protein precursor [*Bombyx mori*] |
| gi|357631806| | 219 | 2.179 | 0.001 | -11.089 | Trypsin-like serine protease [*Danaus plexippus*] |
| gi|114053005| | 214 | 1.115 | 0.001 | -10.123 | Serine protease 7 precursor [*Bombyx mori*] |
| gi|357616527| | 488 | 0.489 | 0.001 | -8.933 | Serine protease [*Danaus plexippus*] |
| gi|114051802| | 1125 | 44.532 | 6.091 | -2.87 | Chymotrypsin-like serine protease precursor [*Bombyx mori*] |
| gi|289722652| | 207 | 32.27 | 5.518 | -2.548 | Trypsin-like serine protease precursor [*Glossina morsitans*] |
| gi|347810668| | 220 | 10.844 | 2.077 | -2.385 | Trypsin-like serine proteinase 1 [*Plutella xylostella*] |
| gi|357625372| | 1063 | 46.232 | 11.604 | -1.994 | Putative serine protease [*Danaus plexippus*] |
| gi|301153729| | 854 | 10.336 | 2.675 | -1.95 | Trypsin-like serine proteinase 1 [*Plutella xylostella*] |
| gi|237700780| | 249 | 175.332 | 50.456 | -1.797 | Serine protease 7 [*Mamestra configurata*] |
| gi|62082417| | 266 | 41.256 | 12.881 | -1.679 | Trypsin-like serine protease [Ostrinia nubilalis] |
| gi|239050264| | 641 | 39.079 | 13.185 | -1.567 | Serine protease homolog 42 isoform 1 precursor [*Nasonia vitripennis*] |
| gi|357623816| | 321 | 8.175 | 2.846 | -1.522 | Transmembrane serine protease 9 [*Danaus plexippus*] |
| gi|229619532| | 628 | 81.295 | 29.099 | -1.482 | Silk gland derived serine protease [*Bombyx mori*] |
| gi|304443623| | 1027 | 1511.31 | 560.72 | -1.43 | Serine protease 13 [*Mamestra configurata*] |
| gi|357611663| | 222 | 25.791 | 10.289 | -1.326 | Serine protease precursor [*Danaus plexippus*] |
| gi|315139348| | 871 | 1206.25 | 514.29 | -1.23 | Serine protease 62 [*Mamestra configurata*] |
| gi|112982842| | 627 | 0.761 | 0.364 | -1.063 | Clip domain serine protease 4 precursor [*Bombyx mori]* |
| gi|237700823| | 225 | 34.99 | 17.259 | -1.02 | Serine protease 5 [*Mamestra configurata*] |
| gi|357606581| | 277 | 0.001 | 0.825 | 9.688 | Serine protease inhibitor [*Danaus plexippus*] |
| gi|112984548| | 278 | 3.433 | 16.434 | 2.259 | Serine protease inhibitor 5 precursor [*Bombyx mori*] |
| gi|112982980| | 1767 | 43.474 | 175.68 | 2.015 | Serine protease inhibitor 4 precursor [*Bombyx mori*] |
| gi|33590491| | 462 | 118.767 | 977.98 | 3.042 | Serine proteinase inhibitor [*Procambarus clarkii*] |
| gi|117970186| | 312 | 29.821 | 158.87 | 2.413 | PxSerpin 3 [*Plutella xylostella*] |
| gi|357622451| | 219 | 26.144 | 103.26 | 1.982 | Serine protease inhibitor 11 [*Danaus plexippus*] |
| gi|226342886| | 1501 | 7.788 | 23.132 | 1.571 | Serine protease inhibitor 13 precursor [*Bombyx mori*] |
| gi|226342884| | 980 | 57.938 | 160.83 | 1.473 | Serine protease inhibitor 11 precursor [*Bombyx mori*] |
| gi|357601811| | 418 | 260.25 | 553.03 | 1.087 | Serine protease inhibitor 28 [*Danaus plexippus*] |
| gi|195972056| | 342 | 5.581 | 11.355 | 1.024 | Serpin-27 [*Bombyx mori*] |
| gi|117970183| | 630 | 7.252 | 20.449 | 1.496 | PxSerpin 2 [*Plutella xylostella*] |
| gi|357611911| | 249 | 5.749 | 2.752 | -1.063 | Serpin-27 [*Danaus plexippus*] |
| ***Effect System*** | | | | | |
| gi|117970197| | 1347 | 9.387 | 130.917 | 3.802 | PxProphenoloxidase-activating proteinase 3 [*Plutella xylostella*] |
| gi|294846061| | 1679 | 4.121 | 24.625 | 2.579 | Prophenoloxidase activating proteinase 1 [*Biston betularia*] |
| gi|25989211| | 872 | 41.038 | 221.88 | 2.435 | Prophenoloxidase activating factor 3 [*Bombyx mori*] |
| gi|35277829| | 1461 | 54.702 | 130.71 | 1.257 | Prophenoloxidase-activating proteinase-3 precursor [*Manduca sexta*] |
| gi|294846061| | 1652 | 0.289 | 0.001 | -8.174 | Prophenoloxidase activating proteinase 1 [*Biston betularia*] |
| gi|281022082| | 409 | 9.387 | 130.917 | 3.802 | Antibacterial peptide cecropin 1 [*Plutella xylostella*] |
| gi|117970169| | 210 | 553.25 | 2233.1 | 2.013 | PxCecropin E [*Plutella xylostella*] |
| gi|313247982| | 342 | 0.6976 | 2.6717 | 1.937 | Cecropin 1 [*Helicoverpa armigera*] |
| gi|223006754| | 896 | 435.6 | 1919.2 | 2.139 | Gloverin [*Plutella xylostella*] |
| gi|357605426| | 210 | 0.001 | 4.351 | 12.087 | Putative lysozyme [*Danaus plexippus*] |
| gi|357611488| | 315 | 0.001 | 0.725 | 9.502 | I-type lysozyme [*Danaus plexippus*] |
| gi|153791889| | 1154 | 0.001 | 0.792 | 9.629 | lysozyme-like protein 1 precursor [*Bombyx mori*] |
| **Xenobiotics detoxification** | | | | | |
| gi|87082475| | 214 | 0.001 | 4.270 | 12.060 | Cytochrome P450 CYP314A1 [*Manduca sexta*] |
| gi|161344535| | 225 | 0.001 | 3.046 | 11.573 | Cytochrome P450 CYP6AE9 [*Bombyx mandarina*] |
| gi|158323895| | 329 | 0.001 | 2.083 | 11.024 | Cytochrome P450 [*Plutella xylostella*] |
| gi|291464079| | 736 | 0.001 | 1.552 | 10.600 | Cytochrome P450 4CG1 [*Manduca sexta*] |
| gi|158323899| | 234 | 0.001 | 0.976 | 9.931 | Cytochrome P450 [*Plutella xylostella*] |
| gi|357631567| | 303 | 0.001 | 0.754 | 90558 | Putative cytochrome P450 [*Danaus plexippus*] |
| gi|81248546| | 336 | 0.001 | 0.680 | 9.409 | Cytochrome P450 [*Helicoverpa armigera*] |
| gi|357621279| | 362 | 0.001 | 0.631 | 9.302 | Cytochrome P450 6AB4 [*Danaus plexippus*] |
| gi|291464091| | 2230 | 0.214 | 5.941 | 4.800 | Cytochrome P450 4G49 [*Manduca sexta*] |
| gi|357614194| | 1641 | 0.145 | 0.001 | -7.184 | Putative cytochrome P450 [*Danaus plexippus*] |
| gi|163256092| | 765 | 57.381 | 11.944 | -2.264 | Cytochrome P450 [*Plutella xylostella*] |
| gi|291464075| | 1134 | 1.893 | 0.403 | -2.233 | Cytochrome P450 4M1 [*Manduca sexta*] |
| gi|84993716| | 322 | 14.818 | 3.547 | -2.062 | Cytochrome P450 [*Plutella xylostella*] |
| gi|357601904| | 349 | 2.734 | 0.655 | -2.063 | Cytochrome P450 [*Danaus plexippus*] |
| gi|49532926| | 803 | 0.907 | 37.34 | 5.37 | Glutathione S-transferase 2 [*Plutella xylostella*] |
| gi|363896092| | 383 | 27.41 | 94.23 | 1.78 | UDP-glycosyltransferase [*Helicoverpa armigera*] |
| gi|363896080| | 764 | 12.49 | 37.67 | 4.59 | UDP-glycosyltransferase [*Helicoverpa armigera*] |
| gi|357618336| | 493 | 16.45 | 44.94 | 1.45 | UDP-glycosyltransferase [*Helicoverpa armigera*] |
| gi|363896116| | 1792 | 64.83 | 114.81 | 1.16 | UDP-glycosyltransferase [*Helicoverpa armigera*] |
| gi|357618207| | 347 | 15.81 | 1.36 | -3.59 | Aldehyde oxidase 2 [*Danaus plexippus*] |
| gi|357630693| | 1403 | 100.32 | 19.05 | -2.40 | Aldehyde oxidase 2 [*Danaus plexippus*] |
| gi|357607768| | 1981 | 189.72 | 42.18 | -2.17 | Aldehyde oxidase 1 [*Danaus plexippus*] |
| gi|294846800| | 1331 | 8.78 | 2.92 | -1.59 | Carboxyl/choline esterase [*Helicoverpa armigera*] |
| gi|294846812| | 860 | 46.05 | 19.12 | -1.27 | Carboxyl/choline esterase [*Helicoverpa armigera*] |
| **Apoptosis** | | | | | |
| gi|343796623| | 921 | 18.65 | 55.56 | 1.57 | Serine/threonine protein kinase Akt [*Helicoverpa armigera*] |
| gi|357624008| | 1399 | 21.15 | 53.88 | 1.35 | Inhibitor of nuclear factor kappa B kinase beta subunit [*Danaus plexippus*] |
| gi|321400082| | 655 | 2.79 | 14.57 | 2.38 | Apoptosis-inducing factor [*Bombyx mori*] |
| **Calcium signaling pathway** | | | | | |
| gi|56462266| | 638 | 189.21 | 44.04 | -2.10 | Troponin C 2 [*Lonomia obliqua*] |
| gi|225711400| | 412 | 45.17 | 11.09 | -2.03 | Calmodulin [*Caligus rogercresseyi*] |
| gi|357625891| | 1078 | 26.34 | 8.46 | -1.64 | Calmodulin-A [*Danaus plexippus*] |
| gi|357627331| | 111 | 28.19 | 8.18 | -1.79 | Mitochondrial ADP/ATP carrier protein [*Danaus plexippus*] |
| gi|4191598| | 1875 | 29.26 | 10.84 | -1.43 | Plasmic reticulum-type calcium ATPase [*Heliothis virescens*] |
| gi|256773186| | 1851 | 109.94 | 41.34 | -1.41 | Plasmic reticulum calcium ATPase [*Bombyx mori*] |
| gi|357622614| | 912 | 2.38 | 0.93 | -1.36 | Stretchin-Mlck, isoform A [*Danaus plexippus*] |
| gi|356470637| | 1186 | 21.72 | 9.24 | -1.23 | Ryanodine receptor [*Plutella xylostella*] |
| gi|354463161| | 1841 | 17.88 | 8.44 | -1.08 | Ryanodine receptor 2 [*Plutella xylostella*] |
| **Insect hormone biosynthesis** | | | | | |
| gi|112983178| | 1293 | 21.21 | 4.24 | -2.32 | Juvenile hormone esterase 1 precursor [*Bombyx mori*] |
| gi|357607885| | 1066 | 12.08 | 4.29 | -1.50 | Juvenile hormone epoxide hydrolase [*Danaus plexippus*] |
| gi|357622740| | 1613 | 16.86 | 6.94 | -1.29 | Juvenile hormone epoxide hydrolase [*Danaus plexippus*] |
| gi|156619502| | 1889 | 31.83 | 9.92 | -1.68 | Carboxylesterase [*Helicoverpa armigera*] |
| gi|383510913| | 1008 | 19.17 | 6.57 | -1.54 | Carboxylesterase [*Helicoverpa armigera*] |
| **Others** | | | | | |
| gi|357617949| | 9 | 0.657 | 5.663 | 3.107 | Hemolymph proteinase 9 [*Danaus plexippus*] |
| gi|357629575| | 113 | 6.435 | 49.734 | 2.950 | Putative hemolymph proteinase 5 [*Danaus plexippus*] |
| gi|56418391| | 48 | 8.482 | 48.731 | 2.522 | Hemolymph proteinase 5 [*Manduca sexta*] |
| gi|56418397| | 842 | 58.151 | 300.524 | 2.370 | Hemolymph proteinase 8 [*Manduca sexta*] |
| gi|56418399| | 14 | 2.945 | 13.160 | 2.160 | Hemolymph proteinase 9 [*Manduca sexta*] |
| gi|198278344| | 429 | 0.001 | 0.532 | 9.057 | Laccase 2A [*Bombyx mori*] |
| gi|357611899| | 258 | 1.849 | 17.707 | 3.259 | Laccase 1 [*Danaus plexippus*] |
| gi|26190489| | 593 | 7.644 | 31.202 | 2.029 | Laccase 1 [*Manduca sexta*] |
| gi|20384700| | 200 | 0.001 | 1.142 | 10.158 | Acetylcholinesterase [*Plutella xylostella*] |
| gi|148298780| | 580 | 0.001 | 1.182 | 10.206 | Carboxypeptidase [*Bombyx mori*] |
| gi|225718942| | 289 | 4.128 | 0.001 | -12.011 | Carboxypeptidase B [*Caligus clemensi*] |
| gi|357610307| | 349 | 2.051 | 0.001 | -11.002 | Cadherin [*Danaus plexippus*] |
| gi|357613568| | 332 | 15.809 | 2.752 | -2.522 | Hdd1-like protein [*Danaus plexippus*] |
