# Supplementary material for: Transcript and Protein Profiling Analysis of the Destruxin A-Induced Response in Larvae of Plutella xylostella
Source: PLoS One. 2013 Apr 9;8(4):e60771. doi: 10.1371/journal.pone.0060771 (PMC3621956; doi:10.1371/journal.pone.0060771)
Supplement: Table S2 — Identification of hemolymph proteins by MALDI-TOF/TOF-MS/MS. (DOC) [file pone.0060771.s002.doc]

**Table S2. Identification of hemolymph proteins showing differential expression after the treatment of destruxin A for 4 hour using MALDI-TOF/TOF-MS/MS analysis.**

| Spot | Fold | Accession number (NCBI) | Protein name | Calculated pI value | Nominal mass (Mr) Da | Mascot score |
| --- | --- | --- | --- | --- | --- | --- |
| 1 | -100000 | gi|114842153 | Arylphorin-like hexamerin-2 [*Plutella xylostella*] | 5.5 | 83011 | 186 |
| 2 | -41.9391 | gi|114842151 | Arylphorin-like hexamerin-1 [*Plutella xylostella*] | 5.99 | 82627 | 203 |
| 3 | -100000 | gi|114842153 | Arylphorin-like hexamerin-2 [*Plutella xylostella*] | 5.5 | 83011 | 214 |
| 4 | -11.1396 | gi|114842153 | Arylphorin-like hexamerin-2 [*Plutella xylostella*] | 5.5 | 83011 | 236 |
| 5 | -100000 | gi|114842151 | Arylphorin-like hexamerin-1 [*Plutella xylostella*] | 5.99 | 82627 | 125 |
| 6 | -30.6618 | gi|114842153 | Arylphorin-like hexamerin-2 [*Plutella xylostella*] | 5.5 | 83011 | 198 |
| 7 | -2.2722 | gi|114842151 | Arylphorin-like hexamerin-1 [*Plutella xylostella*] | 5.99 | 82627 | 180 |
| 8 | -100000 | gi|114842153 | Arylphorin-like hexamerin-2 [*Plutella xylostella*] | 5.5 | 83011 | 200 |
| 9 | -100000 | gi|114842153 | Arylphorin-like hexamerin-2 [*Plutella xylostella*] | 5.5 | 83011 | 203 |
| 10 | -4.0422 | gi|378133017 | Putative transposase [*Escherichia coli* DEC10E] | 9.37 | 8936 | 72 |
| 11 | -100000 | gi|114842153 | Arylphorin-like hexamerin-2 [*Plutella xylostella*] | 5.5 | 83011 | 106 |
| 12 | +3.8494 | gi|22450115 | Glucosinolate sulfatase [*Plutella xylostella*] | 5.22 | 59468 | 413 |
| 13 | +6.7111 | gi|283046836 | ATP synthase subunit beta | 5.21 | 55241 | 423 |
| 14 | +7.0099 | gi|270298186 | Enolase [*Pieris rapae*] | 5.85 | 47260 | 158 |
| 15 | +7.0872 | gi|114842153 | Arylphorin-like hexamerin-2 [*Plutella xylostella*] | 5.5 | 83011 | 124 |
| 16 | +100000 | gi|114842151 | Arylphorin-like hexamerin-1 [*Plutella xylostella*] | 5.99 | 82627 | 163 |
| 17 | +100000 | gi|117970173 | PxS-adenosyl-L-homocysteine hydrolase [*Plutella xylostella*] | 5.83 | 47897 | 251 |
| 18 | +100000 | gi|117970173 | PxS-adenosyl-L-homocysteine hydrolase [*Plutella xylostella*] | 5.83 | 47897 | 159 |
| 19 | +3.2520 | gi|68270850 | Actin [*Culex pipiens*] | 5.3 | 42068 | 320 |
| 20 | +100000 | gi|157122933 | Actin [*Aedes aegypti*] | 5.22 | 42149 | 244 |
| 21 | +3.0452 | gi|117970183 | PxSerpin 2 [*Plutella xylostella*] | 6.12 | 43329 | 221 |
| 22 | +4.2388 | gi|117970183 | PxSerpin 2 [*Plutella xylostella*] | 6.12 | 43329 | 223 |
| 23 | +2.6056 | gi|117970183 | PxSerpin 2 [*Plutella xylostella*] | 6.12 | 43329 | 86 |
| 24 | +100000 | gi|117970183 | PxSerpin 2 [*Plutella xylostella*] | 6.12 | 43329 | 347 |
| 25 | +27.6345 | gi|161088166 | Arginine kinase [*Bicyclus anynana*] | 6.78 | 22728 | 274 |
| 26 | +50.3009 | gi|126567522 | Serpin 1b [*Plutella xylostella*] | 5.1 | 43413 | 110 |
| 27 | +7.0299 | gi|357608189 | Hypothetical protein KGM_10825 [*Danaus plexippus*] | 6.4 | 46700 | 176 |
| 28 | +45.7802 | gi|118424553 | Cathepsin L-like cysteine proteinase [*Spodoptera exigua*] | 6.39 | 38747 | 182 |
| 29 | +2.1584 | gi|117970183 | PxSerpin 2 [*Plutella xylostella*] | 6.12 | 43329 | 372 |
| 30 | +100000 | gi|114842151 | Arylphorin-like hexamerin-2 [*Plutella xylostella*] | 5.5 | 83011 | 325 |
| 31 | +100000 | gi|71993870 | Protein Y43F4B.5 [*Caenorhabditis elegans*] | 5.82 | 66659 | 60 |
| 32 | +2.8873 | gi|114050901 | 14-3-3 protein zeta [*Bombyx mori*] | 4.9 | 28266 | 149 |
| 33 | +100000 | gi|158999406 | Trypsin-like enzyme [*Plutella xylostella*] | 4.41 | 29512 | 80 |
| 34 | +155.828 | gi|158999406 | Trypsin-like enzyme [*Plutella xylostella*] | 4.41 | 29512 | 182 |
| 35 | +4.2946 | gi|114842151 | Arylphorin-like hexamerin-1 [*Plutella xylostella*] | 5.99 | 82627 | 53 |
| 36 | +12.1291 | gi|49532926 | Glutathione S transferase 2-like protein [*Plutella xylostella*] | 5.85 | 23578 | 198 |
| 37 | +96.6243 | gi|398968749 | Hypothetical protein PMI25_ 04229 [*Pseudomonas* sp. GM30] | 6.06 | 11006 | 93 |
| 38 | +14.4406 | gi|387880188 | Hypothetical protein Spaf_1718 [*Streptococcus parasanguinis*] | 5.43 | 22482 | 88 |
| 39 | +4.5172 | gi|346471355 | Hypothetical protein [*Amblyomma maculatum*] | 6.84 | 17214 | 156 |
| 40 | +4.6595 | gi|153792659 | Actin-depolymerizing factor 1 [*Bombyx mori*] | 6.17 | 17227 | 203 |
| 41 | +100000 | gi|346471355 | Hypothetical protein [*Amblyomma maculatum*] | 6.84 | 17214 | 172 |
| 42 | +5.4039 | gi|114842151 | Arylphorin-like hexamerin-1 [*Plutella xylostella*] | 5.99 | 82627 | 314 |

Note: Spot number corresponds to the number of protein spots in Fig 3. Theoretical molecular weight (Mr) and isoelectric point (pI) of the identified proteins were retrieved from the protein database of NCBInr. Experimental Mr and pI were calculated with PDQuest Software. Mascot score is search against the database NCBInr. Protein name is given when proteins were identified by MALDI-TOF/TOF-MS/MS. Accession number is the unique number given to mark the entry of a protein in the database NCBInr.
